# Topical biomaterials to prevent post-tonsillectomy hemorrhage

**DOI:** 10.1186/s40463-019-0368-1

**Published:** 2019-09-06

**Authors:** Lumei Liu, Cole Rodman, Noah E. Worobetz, Jed Johnson, Charles Elmaraghy, Tendy Chiang

**Affiliations:** 1 Center of Regenerative Medicine, Research Institute at Nationwide Children’s Hospital, Columbus, OH USA; 2 College of Medicine, The Ohio State University, Columbus, OH USA; 3 Department of Pediatric Otorhinolaryngology, Nationwide Children’s Hospital, Columbus, OH USA; 4 Nanofiber Solutions, Hilliard, OH USA

**Keywords:** Post-tonsillectomy hemorrhage, Biomaterial, Topical, Surgical hemostasis, Tonsillectomy

## Abstract

Despite advances in surgical technique, postoperative hemorrhage remains a common cause of mortality and morbidity for patients following tonsillectomy. Application of biomaterials at the time of tonsillectomy can potentially accelerate mucosal wound healing and eliminate the risk of post-tonsillectomy hemorrhage (PTH). To understand the current state and identify possible routes for the development of the ideal biomaterials to prevent PTH, topical biomaterials for eliminating the risk of PTH were reviewed. Alternative topical biomaterials that hold the potential to reduce the risk of PTH were also summarized.

## Background

Tonsillectomy is among the most common surgeries performed in pediatric patients. Tonsillectomy is primarily indicated for children who suffer from recurrent infection of the tonsils and / or for tonsillar enlargement that contributes to airway obstruction and sleep disturbance. Over 530,000 children under the age of 15 undergo tonsillectomy annually in the United States [1–3]. Following surgical removal of the tonsils, the surgical site(s) (tonsillar fossae) heal secondarily. One of the most common complications following tonsillectomy is hemorrhage from the tonsillar fossae, called post-tonsillectomy hemorrhage (PTH) [4]. This can either occur within the first 24 h (primary PTH) or from > 24 h to 2 weeks after surgery (secondary PTH). PTH rates (2.5–7%) have been increasing according to recent statistics: there are an estimated 25,000 cases of PTH in the United States each year, while in 1995 that number was 4300 [5]. Post-tonsillectomy hemorrhage often requires hospital readmission, surgical intervention, and can even result in significant morbidity such as shock, airway obstruction and need for blood transfusion [6]. The hemorrhage is generally unexpected and unpredictable [7–12], and resuscitation with life-threatening PTH can prove especially challenging [13, 14].

Attempts to reduce the morbidity of tonsillectomy have included changes to the surgical approach and peri-operative medications to reduce the risk of bleeding. Technical modifications to the procedure, such as partial or intracapsular tonsillectomy, can reduce pain and PTH [15–17]. However, such modifications are not indicated for chronic infection and can result in tissue regrowth, requiring revision surgery [18, 19].

A low cost, easily applied, effective strategy to prevent PTH remains elusive. Topical biomaterials have many appealing properties, making them a potentially ideal strategy for PTH prevention. Biomaterials can be fabricated with adhesive properties, and once adherent, they provide physical barriers to protect healing tissue from complex shear forces created by cough and swallowing. Some biomaterials can confer antimicrobial properties which facilitate the PTH prevention by protecting tonsil wounds from infection. An ideal topical biomaterial can prevent PTH by accelerating wound healing and remucosalization, and by expediting and amplifying the coagulation cascade and clot formation. Additionally, a topical therapy can include analgesic substances to locally reduce pain.

The proposed mechanism of secondary hemorrhage is retraction and sloughing of the eschar covering the healing tonsillar bed [20]. The tonsillar bed is at especially high risk for secondary hemorrhage due to the complexity of the tonsil environment, the lack of self-compression by surrounding tissues, and the robust vascular supply to the area. Thus, wound healing and clot stabilization is a critical process in preventing PTH. Biomaterials preventing PTH through improving wound healing after tonsillectomy have been poorly reported. Many studies evaluated surgical outcomes of biomaterials including pain, hemorrhage severity, and incidence of bleeding, but there are few standard methods to evaluate their impact on wound healing after tonsillectomy [21]. An ideal, functional biomaterial is expected to 1) facilitate hemostasis intraoperatively by improving coagulation and clot formation; 2) stabilize the clot and provide protection or compression within the first 24 h, thus preventing primary PTH; 3) improve wound healing and remucosalization, shortening the at-risk time from secondary PTH; 4) provide a physical barrier for the duration of the wound healing process.

Since the mid-90s’, numerous studies have been devoted to the study of topical biomaterials (sulfathiazole gum) to prevent PTH [22]. We present a comprehensive review of these topical biomaterials, categorizing them into fibrin-based, gelatin-based, cellulose-based, bismuth subgallate-based, antifibrinolytics, and natural materials. We also include other promising biomaterials as potential target therapies for PTH prevention. These biomaterials have specific properties to prevent PTH to some degree, however, some limitations and side effects impeded their application in reality, for example high cost (fibrin- based patch), inconsistent clinical outcomes (fibrin-based sealant and gelatin-based matrix), risk of suffocation (cellulose-based hemostats), non-reduced pain (cellulose-based hemostats secured with suture) etc. We hope to illuminate the current state of topical methods of PTH prevention and offer a course of future directions to develop biomaterial for PTH prevention.

## Main text: topical biomaterials used for PTH

### Search strategy

All topical biomaterials used for PTH were searched in databases of Google Scholar and the Food and Drug Administration (FDA). The key words used to identify related topics include: post-tonsillectomy hemorrhage, biomaterial, topical, hemostasis, tonsillectomy. Articles and patents published and searchable in google scholar after the year of 2000 were reviewed. The FDA approval status of identified biomaterials for PTH were found in FDA website. The reference lists of all relevant primary studies, review articles and patents.

#### Fibrin-based biomaterials

Fibrin is a common biomaterial that has been widely applied as a hemostatic sealant in clinical and bioengineering fields [23–26]. The hemostasis and wound healing mechanism of fibrin-based biomaterial follows the principles of physiological fibrin clot formation. Most fibrin-based biomaterials contain fibrinogen and thrombin (Quixil® does not contain fibrinogen). Thrombin (a clotting enzyme) transforms fibrinogen to fibrin monomers when combined with calcium and factor XIII from wound surfaces. This initiates polymerization of fibrin monomers to polymeric fibrin clots. Fibrin clots are large mesh-like polymers that trap platelets and red blood cells, which in turn release more clotting factors amplifying the cycle. When procedures for wound closure are limited, clots are relied upon to achieve tissue sealing and hemostasis in several surgical procedures [27]. Thrombin (Factor IIa) initiates cleavage of fibrinogen into fibrin monomers, accelerating clot formation [28, 29]. Meanwhile thrombin activates factor XIII, which stabilizes coagulated fibrin by covalently crosslinking [30]. Thrombin also acts directly on platelets, factors V, VII, and X, XII, and smooth muscle cells, leading to hemostasis and wound healing [31, 32].

Current forms of fibrin-based sealant include liquid fibrin-based glue and a fleece/patch. The first fibrin-based glue product approved by Food and Drug Administration (FDA) was Tisseel® fibrin sealant glue (Baxter International, Deerfield, IL) [33]. Later, fibrin glue products such as Tissucol® (Baxter International) and Quixil® (OMRIX biopharmaceuticals, Inc., New York City, NY) were used in tonsillectomy patients [34, 35]. Application of these fibrin glues had no effect on post-tonsillectomy pain control and bleeding prevention [34, 35]. In a review of hemostatic glues in tonsillectomy, it was concluded that fibrin glues were not recommended for routine use in current clinical practice as they were not effective in reducing severity of pain and bleeding [31]. However, other studies showed beneficial outcomes of fibrin-based glue for PTH prevention. Quixil tested in a prospective randomized study reduced pain and prevented bleeding in tonsillectomy patients compared with bipolar or needle point electrocautery (0% vs. 4.35%) [36]. Another prospective randomized single-blind study proved Quixil can decrease immediate inflammatory response in patients after tonsillectomy [36, 37]. In a prospective randomized study of 40 patients, another liquid form of fibrin sealant (spray) reduced pain compared with electrocautery hemostasis alone [38]. Evicel® (Ethicon Inc., Somerville, NJ) is a new formulation of previously available fibrin sealant Quixil (EU) or Crosseal™ (US), and it has been used as an effective hemostat in orthopedic [39], endonasal surgeries [40, 41], and epistaxis management [42, 43] but benefit in tonsillectomy has yet to be assessed.

Compared with fibrin glue, fibrin-based patch or fleece has demonstrated advantages to be used for PTH prevention. Compared to the complex manipulation of fibrin glue, which requires storage at low temperatures and long thawing time [31], fibrin-based patches are ready-to-use. They do not require preparation, mixing, moistening with saline or refrigeration. Another reason fibrin-based glues are not ideal PTH prevention is that they lack the necessary mechanical strength to perform as an adhesive or tissue sealant [44]. A patch/fleece provides a robust and solid mechanical support to fibrin glue, making the fibrin-based patch an effective sealant. For example, one fibrin-based fleece product, TachoComb® (Nycomed Pharma, Switzerland) is comprised of a collagen sponge and a dried layer of fibrinogen and thrombin. TachoComb is a widely used method that has been used in diffuse bleeding from parenchymatous organs or bleeding of the lung both in conventional and endoscopic surgery [45]. One prospective study was performed to assess TachoComb in children for postoperative complications after tonsillectomy (*n* = 53 for treatment group and *n* = 57 for control group). It was demonstrated the fibrin-based patch significantly reduced pain and post-surgery bleeding from 8.8 to 0%. [46]. While in another prospective study, the rate of postoperative hemorrhage and re-admission for PTH in TachoComb treatment group were not significant different with control group [47].

Compared with TachoComb, TachoSil® (Takeda Pharmaceutical Company, Tokyo, Japan) is an FDA approved biomaterial that is non-aprotinin and contains human thrombin instead of the bovine thrombin found in TachoComb [47–49]. Common ingredients in both TachoSil and TachoComb patches is human fibrinogen (5.5 mg/cm^2^), and additional active ingredients in TachoComb is bovine aprotinin [50], which could explain different outcome in future comparative studies. TachoSil is recommended for use in small wounds due to the poor sealant properties in large wounds [51]. TachoSil has been used in a variety of abdominal surgeries including hepatic, pancreatic, spleen, gastrointestinal, and inguinal hernia repair [52]. However, it was shown that TachoSil has a risk of adhesive failure compared with another fibrin sealant patch, Evarrest® (Ethicon), in a swine spleen incision model [53]. Evarrest has been approved by FDA [54], and has been used to treat severe soft tissue surgical bleeding such in abdominal, pelvic, and retroperitoneal sites, and in non-cardiac thoracic, liver, cardiovascular surgeries [55–57]. Although Evarrest has not been tested with tonsillectomies, its application in these surgeries and relevant results would encourage the use of Evarrest for PTH.

Some fibrin-based biomaterial products contain tranexamic acid (TXA) instead of aprotinin as an antifibrinolytic adjuvant. Of note, TXA is potentially neurotoxic [41]. In 2010, TachoComb was evaluated in pigs that the aprotinin component caused anaphylactic relations and risk of renal failure [47]. It was also too expensive for some surgical units [58]. The high cost of fibrin-based patches might limit the clinical application of this biomaterial [59, 60]. Despite improvement in PTH rates demonstrated in Quixil, TachoComb, TachoSil, and Evarrest, application has not been widely adopted due to 1) Lack of persistence for entire bleeding risk interval; 2) Analphylaxis; 3) Attachment failure; 4) Not tested yet; 5) High cost.

#### Gelatin-based biomaterials

Gelatin-thrombin hemostatic matrix (GTHM) agent is a gelatin-based biomaterial consisting of bovine-derived gelatin matrices and human-derived thrombin. It has been used to treat and manage secondary PTH [61]. Gelatin-based materials provide a combination of two independent hemostatic agents, gelatin and thrombin, in order to achieve hemostasis [62, 63]. Gelatin matrix granules swell to produce a tamponade effect for wound healing [28, 64]. Upon delivery to the wound site, it serves as a substrate for platelet adhesion and fibrin activation, leading to clot formation. Thrombin accelerates and stabilizes fibrin clot formation, improving hemostasis and wound healing [31, 32].

The absorbable liquid form product (Table 1), FloSeal™ Hemostatic Matrix (Baxter International) was approved by the FDA [72]. FloSeal has been advocated to improve intraoperative hemostasis in cardiac and spinal surgeries [63, 90], transphenoidal pituitary surgery [91], the management of epistaxis [92], endoscopic sinus surgery [93], and is commonly used in otolaryngologic interventions by otolaryngologists [94]. FloSeal was tested in a randomized controlled trial on 68 pediatric patients undergoing adenotonsillectomy. Results showed that Floseal was safe and efficacious, and decreased postoperative morbidity and blood loss comparing with electrocautery hemostasis after cold steel adenotonsillectomy [67]. In a later clinical trial, FloSeal showed reduction in pain medication, promoted mucosal recovery and faster wound healing (less thickness of wound plaques), but no significant reduction in postoperative hemorrhage [69]. Another study concluded that Floseal on ligatured fossa had no effect on pain reduction comparing with control group (ligatured fossa without Floseal) [68]. Absorbable gelatin medicated sponges or powders [95, 96] (Table 2) are another type of gelatin-based hemostat, which have not been used for PTH yet. They are discussed in section two.

**Table 1:** Summary of topical biomaterials to prevent PTH

Biomaterial		Decrease PTH severity (Y/N)	Decrease PTH incidence (Y/N)	Reduce pain (Y/N)	Study type	Commercial Products	Administer type	Other outcomes	FDA approval for human (Y/N)
Fibrin-based Hemostasis glue/fleece/ patch/	Fibrin glue: Fibrinogen and/or thrombin	N [31, 34, 35]	Y [36]	N [31, 34, 35], Y (spray) [38]	randomized double-blind study on 50 adults patients [34], prospective randomized double-blind study on 168 consecutive patients and systematic review [31]	Tissucol™/ Tisseel™, and Crosseal™ / Quixil®, Evicel®	Post-op	Easy to use, maybe time efficient in operation but storage at low temperatures, and long thawing time [31]. decreased immediate inflammatory response following adenotonsillectomy [37]. Not enough sample sizes were studied [31].	Y [59, 65]
	Fibrin-based collagen fleece/patch	reduced emergency surgery for severe PTH without an apparent adverse effect [66]	N [66]	Y [66]	Clinical study on 1057 children patients [66]	TachoComb®, TachoSil®, Evarrest® fibrin sealant patch	Post-op	provided a mechanical scaffold on which vascular regeneration occurred. Not cost efficient. Too expansive for some surgical units [58]	Y TachoSil [48] Y Evarrest® [54] N TachoComb
Gelatin-based Hemostat	Gelatin-Thrombin Hemostatic Matrix (GTHM)	Y [67] N	Y [61], [67]	N [68]	Case study by reviewing 42 pediatric patients and retrospective data analysis [61], randomized, controlled trial in adults [68, 69] and children [67, 70] and a review [71]	Floseal™ Hemostatic Matrix	Post-op [61, 71]	simple, safe, and efficacious and cost-effective [61, 70]	Y [72]
Absorbable cellulose-based Hemostat	oxidized cellulose polymer hemostatic agent	Y [73]	Y (when combine with suturation) [74]	N (when combine with suturation) [74]	Surgicel plus saturation on total of 760 patients (393 males, 367 females) between the ages of 4 and 35 years [74]	Surgicel®, Cellistypt®, Pahacel®, Oxycel®, Gelita®, GuraTamp®	Intra-op	aids in clot formation by activation of the extrinsic and intrinsic coagulation pathways, Introduced a risk of aspiration and suffocation [75]; Possible inflammation [76].	Y Surgicel® [77] N for others
Bismuth subgallate-based	BSG- adrenaline paste, BSG- phenylephrine hydrochloride mixture	Y [78]	Y (BSG mixed with phenylephrine hydrochloride) [79] Y BSG with adrenaline for primary PTH [80], N for secondary PTH [81]	N [81]	Clinical study on patients underwent tonsillectomy [78, 81]	Spectrum Chemical (powder), mixing 26 g of BSG powder to 20 ml of normal saline with 0.7 ml of 1:1000 adrenaline	Post-op	decreases operating time by significantly reducing the hemostasis time and the number of ligatures [78, 81]; accelerates the cascade of blood clotting [79]; accelerate the intrinsic clotting pathway through the activation of factor XII (Hageman factor) [82].	Y [62]
Antifibrinolytics	tranexamic acid (TXA) liquid	Y [83, 84]	N [83, 84]	–	Clinical reports analysis of 246 patients topically treated with TA and 248 control [84] Systematic review [83]	Cyklokapron®	Postop	no device or site for infection, reduce intraoperative blood loss during Orthognathic Surgery, prevent postop hemorrhage after oral or dental surgery in hemophilia A patients and those on warfarin anticoagulation [85]	N for Topical application
Natural material	Propolis	–	Y [86]	Y [86]	Randomized controlled study on 65 patients [86]	Topical propolis gel	gargle immediately after surgery [86]	accelerated wound healing of tonsillar fossae	N
	Herbal ingredients	Y [87]	–	–	Clinical trial in 47 consecutive children patients	Ankaferd Blood Stopper®	Intra-op	safe and efficient, and it reduces operating time [87], and contains no synthetic additives [88]	N
	Autologous serum	–	Y [89]	Y [89]	A preliminary study on 32 patients (4–15 years old) [89]	Centrifuge peripheral venous blood at 1500–2000 g for 10 min to separate the serum and then topically administered	Intra and post 8 and 24 h	Contributed to tonsillar fossa epithelization in postoperative period Only preliminary study was performed	N

**Table 2:** Alternative biomaterials applied in other hemorrhage applications with potential for PTH

Biomaterial		Commercial Product	Mechanism of action	Application	Advantages	Drawbacks	Action/ resorption time	FDA approval (Y/N)
Scaffold/Matrix Agents	Absorbable gelatin medicated sponge [95, 96] (sponge or powder)	Gelfoam®, Surgifoam®	porous matrix for platelet adhesion and fibrin clot formation; increased volume can provide hemostasis [29]	otosurgery [97], cardiac [63], lumbar spine [98]	little allergic activity [29]	Increased granuloma and infection [29]	4–6 weeks	Y [99, 100]
	microfibrillar collagen-based powder, sheets, or plugs	Colgel®, Helitene®, Avitene®, CollaPlug®, CollaTape®	bovine collagen provides a substrate that activates platelets and allows them to adhere, facilitate clot formation through the intrinsic coagulation pathway [29, 101]	Tonsil bleeding [102], epistaxis [103], cardiac surgery [104, 105]	does not swell [29]	Adhesions, foreign body reactions, or allergic reactions have been reported. Most often with Avitene [106].	8 weeks	Y [107–109] except Colgel
	microfibrillar collagen-based atraumatic, bioresorbable, liquid hemostat spray	CoStasis®	combination of bovine collagen and bovine thrombin in a calcium chloride buffer and autologous obtained plasma that are mixed in equal volumes and administered intraoperatively. Fibrinogen from the patient’s plasma is cleaved by the thrombin in the collagen matrix	hepatic orthopedic cardiothoracic, and general surgical procedures [110, 111]	acceptable biocompatibility [112]	Low antigenicity of collagen and possible allergic reactions [113]	achieve hemostasis within 3 min in cardiac surgery [110], 10 min for general surgeries, hepatic and iliac crest surgery [111]	Y [114]
	Microporous mucopolysaccharide spheres	Arista AH®, HemoStase MPH®	flowable potato starch powder engineered to dehydrate blood, enhance clotting, concentrate erythrocytes and platelets for more effective thrombus formation [29, 115]	cardiothoracic surgical procedures [116], cerebral hemostasis [117], nephrectomies [118], sinus [119], cardiac [110]	nonpyogenic, good safety record [29]	short resorption, limited efficacy compared to procoagulant hemostatic [29]	24–48 h.	Y [120]
Biologic hemostats	Topical thrombin liquid (spray)	Bovine origin: Thrombin-JMI®, Thrombogen™ Human pooled: Evithrom®, Recombinant Human (rh): Recothrom®	Thrombin initiates cleavage of fibrinogen to fibrin, promoting clot formation [29]	comparative trial in liver resection, spine, peripheral arterial bypass, and dialysis access surgery [121]	RhThrombin reduced bleeding and thrombotic complications and less immunogenic than bovine thrombin [121]	Activation of autoimmune antibodies, causing profound coagulopathy [122], infectious disease concerns [29]	immediate	N bovine origin thrombin Y Evithrom [123] Y Recothrom [124]
	Platelet-rich fibrin membrane	Spin blood at 3000 rpm for 10 min to get fibrin clot: the middle layer between the red corpuscles at bottom and acellular plasma at the top.	Platelet cytokines, growth factors, and cells are entrapped and discharged in fibrin meshwork serving as a resorbable film [125], and platelets trigger blood clot and wound healing [125].	hemostatic material for the treatment of oral lesions [126–128]	Cost-effective, simplified process, minimum blood manipulation and immunological reaction, promotes soft tissue healing	Need proper protocol and quick handling	–	N
	Chitin/chitosan dressings	ChitoFlex®, HemCon®, Celox™	Promote local vasoconstriction and serve as a scaffold for erythrocyte agglutination; physically occlude the wound site; stimulate fibroblast activation and collagen deposition [86] Interacts with red blood cells and platelets directly to for a clot independent of clotting factors [110]	Cardiac [110], common homeostatic dressing for US military and pre-hospital wound dressing. [110], dental [129]	Antimicrobial due to acidic PH [130], efficacy in acquiring hemostasis and promoted initial healing phases [29, 110],nonallergic, nonexothermic, low cost, able to function in a hypothermic environment [110]	requires dressing and substrate to be in contact with wound	immediate to complete wound healing	Y HemoCon® [131, 132] Y Celox™ [131, 133]
	Hemocoagulase liquid	Hemocoagulase agkistrodon	pharmacologic combination of coagulative proteins present in the venom of *Bothrops jararaca* or *Bothrops atrox* that works by directly cleaving fibrinogen to fibrin and activating factor X. Clot is thus formed independent of thrombin and will not break down in response to antithrombin [29, 134]	minor oral surgical procedures (impactions, simple extractions, transalveolar extractions) [135], postextraction bleeding [136]	Proven to significantly decrease postextraction bleeding [29]	Limitation in purchase and preparation	immediate	N
	granular mineral zeolite-based hemostatic agent (powder in a gauze mesh)	QuikClot®	Zeolite absorbs water and concentrates coagulation factor, causes local dehydration with concentration of erythrocytes and platelets as well as activation of factor XII to initiate the coagulation cascade [29, 110, 137]	Cardiac surgeries [110], exsanguinating extremity wound and bleeding [131]	Cost-effective, stable	cannot be left in the wound site due to a foreign body reaction occurring	immediate	Y [131, 138]
	alginate dressing	Silverlon™ Antimicrobial Calcium Alginate Dressing	Dehydration and concentration of erythrocytes and platelets, cationic initiation of coagulation cascade, barrier protection [139]	effective barrier to microbial penetration for moderate to heavy exuding wounds, and other surgical wounds [140]	Able to be removed with water without disrupting the underlying tissue healing	Not effective to handle a high flow of blood	immediate to complete wound healing	Y [141]
Tissue Adhesives	Cyanoacrylates liquid	Omnex®	The sealant polymerizes to form a flexible sealing film, which is adherent to both synthetic material and human tissue in a process that is independent of the patient’s clotting processes.	Cardiothoracic [142], Neurosurgery [143]	Safe, strong, non-toxic, flexible, biocompatible, prevented blood leakage along suture, diminished hemostasis in coagulopathy, reduced time to hemostasis and seal	if closed in a wound, can lead to tissue necrosis and local inflammation; also do not handle the tension on tissues especially well	immediate to wound healing. The seal degrades with time, breaking down into smaller absorbable fragments.	Y [144]
	Polyethylene glycol hydrogel	CoSeal®, DuraSeal®	2-phase application that results in the formation of a hydrogel matrix that becomes cross-linked with local proteins such as collagen [110]	Cardiac [145], lung [146], laparoscopic lymphadenectomy [147], urologic surgery	Found to be effective for urologic and vascular surgery and for CSF leak [29]	swells to 4 times its size	immediate	Y [148, 149]
	Albumin-based–bovine-derived albumin cross-linked by glutaraldehyde	BioGlue®	Mixture of albumin-based-bovine-derived albumin with glyceraldehyde creates strong crosslinks generating a tough hemostatic and adhesive matrix [29].	Cardiac [132], laparoscopic nephrectomy [150], secure hemostasis at cardiovascular anastomoses [151]	hemostatic and barrier protection	hypersensitivity reaction [29], impairs aortic growth [151], nerve tissue injury, cannot be used in pediatric cases as it impairs tissue growth [110].	immediate/indeterminate 20–30 s and reaches bonding strength by 2 min [110]	Y [152]
	Mucosal tissue dressing based on methyl cellulose [153]	US9381270B2Acclarent, Inc., Menlo Park, CA (US)	Certain embodiments provide a biodegradable film or cover ing that serves as a mechanical barrier to reduce pain caused	for reducing or eliminating pain after Surgical procedures related to mucosal tissue tonsillectomy, adenoidectomy, or other pharyngeal operations.	Reduce pain and bleeding, facilitate mucosal tissue healing	Not clinical tested yet	Expected to be dissolved at 14 days and stable for 5 days	N
	Natural polymer based tissue adhesive (polysaccharides or partial hydrolysis derivatives or neutralization salts, chitosan and an alginate, carboxylic acid (acetic acid and lactic acid)) [154]	US20190038798A1 Ronnie Michael Hanes, Union Grove, AL (US); Adele Lamping Hanes, Union Grove, AL (US	intraoperatively applied on the tonsil fossa which is then closed with the adhesive or sutures, used postoperatively as external dressing by application as a gel, thin film device or dry powder, or any methods in combination.	Post – operative application for tonsillectomy or adenoidectomy surgery, internal tissue adhesive for surgery or wound repair, application to a burn or skin donor site. For internal use, an optional treatment to improve resistance of the activated adhesive to body fluids is also described .	Promote healing with enhanced adhesive properties	Not fully tested yet	–	N

The clinical trials of gelatin-based biomaterials for PTH have inconsistent outcomes on pain, hemorrhage severity and incidence. The other side effects of gelatin-based hemostatic agents include 1) A nidus for infection and abscess formation, as they have been reported to potentiate bacterial growth, 2) foreign body reactions and “encapsulation” of fluid, and 3) toxic shock syndrome, which was reported in association with the use of absorbable gelatin-based hemostats in nasal surgery [155]. Though there were no adverse effects of GTHM reported in secondary PTH [61], it should be proved consistently beneficial effect on pain and hemorrhage to be used for PTH.

#### Oxidized cellulose-based biomaterial

Oxidized cellulose-based biomaterials have been used for decades for their hemostatic properties in the control of oozing from broad surfaces. They are prepared by the oxidation of cellulose with nitrogen tetroxides (N_2_O_4_) [156]. Oxidized cellulose-based biomaterials contain no animal or human components, and are fabricated into mesh, gauze, woven strips, fibrillary tufts, and sponges. Oxidized cellulose acts as a hemostatic by providing a physical matrix for blood absorption and platelets adhesion and aggregation [157]. This accelerates the formation of a platelet plug and fibrin clots, improving wound healing and hemostasis. The low pH of the cellulosic acid within the biomaterial contributes to hemostasis by causing localized vasoconstriction and initial denaturation of blood proteins. The caustic pH exhibits immediate antibacterial effects, minimizing the risk of infection [158], while bacteriostatic and bactericidal properties inhibit growth of gram-positive and gram-negative organisms [159]. Additionally, cellulose-based biomaterials are entirely absorbable. The absorption of cellulose-based biomaterial takes 1 week to 4 weeks, depending on the products [113]. For tonsillectomy, commercial products need to be selected for specific application. For example, GuraTamp® can be completely absorbed within 7–14 days and provide a better option than Gelita-Cel® which can be completely biodegraded in 4 weeks.

Commercial absorbable hemostats products include Cellistypt® (B. Braun Medical Ltd., UK), Pahacel® (Effebi Hospital, Italy), Oxycel® (Becton Dickinson, Franklin Lakes, NJ), Gelita® (Gelita Medical GmbH. Germany), GuraTamp® (Jorgensen Lab, Loveland, CO), and Surgicel® (Ethicon). Surgicel is the most used cellulose-based hemostat in the United States. It is a fibrillary, oxidized-cellulose material in a sterile fabric meshwork. It has been widely used in otolaryngology and oral and maxillofacial surgery in order to control intra-osseous hemorrhage [73]. Its related products have had premarket approval from the FDA since 1960 [77]. A review of topical hemostatic agents in 2010 found that Surgicel had been used in otolaryngologic procedures, such as mastoid and endonasal surgeries [60]. For tonsillectomy, Surgicel application plus suturing decreases PTH rates [74].

No oxidized cellulose-based biomaterial products demonstrate strong adhesive properties, leading to a risk of aspiration and suffocation [75], as well as a potential risk of inflammation [76]. To reduce the risk in tonsillectomy, cellulose-based biomaterial must be combined with suturing which can prolong operative time and adversely impact outcomes [74].

#### Bismuth subgallate-based materials

Bismuth subgallate (BSG) is an insoluble compound that has been showed with conflicting results in reducing hemorrhage incidence after tonsillectomy. The mechanism by which BSG achieves hemostasis is by accelerating the intrinsic clotting pathway via activation of Hageman factor XII [82]. After tonsillectomy performed by dissection and ligation without Diathermy, BSG mixed with adrenaline showed a significant decrease in hemostasis time and operating time in 39 patients, compared with 33 patients who did not receive BSG paste [81]. A prospective randomized trial of 60 patients showed that BSG/adrenaline paste reduced operative blood loss and operative time significantly [78]. One review of medical records showed that BSG reduces the incidence of primary tonsillar hemorrhage [80]. The use of BSG and epinephrine mixture has been reported with a low postoperative bleeding rate (4 of 1428 cases) [79]. The BSG-based paste was shown to be a faster and safer hemostat for PTH compared with the average pediatric tonsillectomy without BSG treatment [160, 161]. However, other studies suggest that BSG did not reduce hemorrhage [162, 163] or post-operative morbidity [81]. In the United States, BSG is an active ingredient in Devrom® (internal deodorant), an over-the-counter FDA-approved medicine [62]. The side effects of BSG include temporary darkening of the tongue and risk of foreign body response that may result in acute pneumonia [157, 164]. Due to BSG’s possible hypersensitivity to the substance, hepatic or renal impairment is the caution impeding its application in patients with liver or kidney disease [157].

#### Antifibrinolytics

Antifibrinolytics are synthetic derivatives of the amino acid lysine. They inhibit fibrinolysis through binding to the enzyme plasmin and preventing the conversion of plasminogen to plasmin on the surface of the fibrin [32]. This prevents the lysis of the fibrin clot, which facilitates hemostasis and wound healing. Antifibrinolytics such as bovine aprotinin and TXA were used as additives in other hemostats for clot stabilization [155]. TXA has been proven effective for PTH by intravenous injection and oral medications, but less effective when applied topically [83, 165, 166].

Currently, TXA has been studied as a topical application for PTH and showed promising results in reducing blood loss in PTH, but no significant effect on PTH rate [83, 84]. CYKLOKAPRON® (Pfizer, New York City, NY) injection is a representative TXA commercial product, which was approved by FDA in 1999 for intravenous injection but not for topical use [167]. TXA has been shown to be effective to prevent oral hemorrhage topically [168, 169]. Oral washes every 6 hours, starting peri-operatively and continuing for 7 days, have been used to prevent postoperative bleeding in patients with hemophilia A [170] and on oral anticoagulation [29, 168].

Side effects of TXA include thromboembolic risk in women who are using combination hormonal contraception, or those who have active or an intrinsic risk of thromboembolic disease. TXA has been implicated in cases venous and arterial thrombosis or thromboembolism, as well as cases of retinal artery and retinal vein occlusions [171]. Due to the life-threatening risk, studies on topical application of TXA in tonsillectomy must be developed to elucidate the benefit over intravenous injection for PTH. A large and well-designed, randomized and controlled trial is needed to investigate the risks and benefits in secondary PTH.

#### Natural materials

Propolis is a resinous mixture produced by mixing bee saliva and beeswax with exudate gathered from tree buds, sap flows, or other botanical sources. Propolis (Seoul Propolis Co., Korea) was immediately applied to bilateral tonsil fossae after surgery and then gargled by patients in a randomized study. The results demonstrated the efficacy of propolis in preventing PTH (from 16.9 to 4.6%), reducing pain, and accelerating wound healing of tonsillar fossae [86]. However, propolis is not approved by the FDA yet and only one study of propolis could be found. To confirm the effect, more clinical trials need to be conducted.

Another natural material, Ankaferd Blood Stopper® (Trend Technology Ilac AS, Turkey), is fabricated with herbal ingredients (plant extracts prepared from *Alpinia officinarum*, *Glycyrrhiza glabra*, *Thymus vulgaris*, *Urtica dioica* and *Vitis vinifera*). It was tested in 47 pediatric patients undergoing tonsillectomy by applying Ankaferd blood stopper on right tonsillar fossa. Patient’s served as their own control with the left tonsillar fossa being knot-tied. Compared to the left, the right side was found significant less bleeding (1.57 ± 2.26 ml vs. 14.04 ± 7.23 ml) and operating time (3.19 ± 0.74 min vs. 7.29 ± 2.33 min) [87]. It is safe and effective, and contains no synthetic additives [88]. It has also showed to improve hemostasis in patients with acute anterior epistaxis [172].

Topically administered autologous serum was found contributed to reduce throat pain and hemorrhage rate in 32 post-tonsillectomy patients aged 4 to 15. In this preliminary study, autologous serum was prepared by centrifuging patient’s blood at 1500–2000 g for 10 min. Then it was administered topically to the right tonsillar fossa for 10 min during the operation and at 8, 24 h post-operatively. It was proposed that the hemostatic benefits were driven by epitheliotropic factors in the autologous serum speeding up the healing process [89]. Additionally, the serum has antibacterial properties due to the immunoglobulin G and lysozymes present therein. It has been used for ocular surface disease treatment and intra-articular and intraosseous injections [173, 174].

However, these natural materials have not been adequately studied in the context of PTH. Their influence on PTH severity, morbidity, and side effect profile need to be verified with more clinical studies.

### Alternative biomaterials with potential for PTH

In addition to the aforementioned biomaterials which have been directly studied in PTH prevention, there are countless more biomaterials for achieving hemostasis intraoperatively that merit consideration for PTH prevention. Of the alternative biomaterials, those that could prove useful in preventing PTH can be categorized as scaffold agents, biologic hemostats, or tissue adhesives (Table 2).

#### Scaffold agents

Microporous mucopolysaccharide spheres (MMS) are small, porous spheres of potato starch whose pores serve as small sieves, wicking away liquid from the wound site and concentrating coagulation factors [29]. The product is a powder which is applied intraoperatively and is resorbed in approximately 24 to 48 h [29]. Arista Ah Absorbable Hemostat® (Medafor Inc., Minneapolis, MN) and HemoStase MPH® (Cryolife Inc., Atlanta, GA) are two commercially available products which have been used in cardiac, orthopedic, spinal, and general surgeries [110]. These products using licensed Microporous polysaccharide hemispheres (MPH) technology received premarket FDA approval [120]. They have been shown to be effective in reducing postoperative bleeding in cardiothoracic surgery [116] and endoscopic sinus surgery without negative impact on nasal mucosa healing, pain, and obstruction in the latter [175, 176]. Ease of use, low risk profile, and compatibility with mucosal healing all make MMSs exciting for PTH prevention, but their short duration of action and inferior hemostatic properties represent significant drawbacks.

Some scaffold products belong to gelatin-based biomaterials and act via the same mechanism as hemostats mentioned in section 1. These products include Gelfoam® (Pfizer) and Surgifoam® (Ethicon) (Table 2), and they, and their related products, have been approved by the FDA [99, 100]. Gelfoam is a water-insoluble, off-white, nonelastic, porous, pliable product prepared from purified porcine skin gelatin. The matrix is fully hydrolyzed by approximately 4 to 6 weeks [29]. It has been widely used to stop hemorrhage in surgeries such as otosurgery [97], cardiac [63],and lumbar spinal surgery [98]. The hemostatic properties of gelatin matrices, their durability in the oral cavity, and their relatively low risk profile make them a potentially promising material for PTH prevention.

Microfibrillar collagen also shows promise as an easy-to-use strategy for reducing PTH. Microfibrillar collagen is a bovine collagen product that can be produced in powders, sheets, or plugs. It provides a substrate for platelet adherence and activation, thereby facilitating the formation of the fibrin clot [177]. It takes approximately 8 weeks for full resorption of the material [29]. Commercial products are Colgel® (Laboratorie Interphar, Aubervilliers, France), Helitene® (Integra Lifesciences Corporation, Plainsboro, NJ), Avitene® (Bard Davol, Warwick, RI), CollaPlug® and CollaTape® (Zimmer, Warsaw, IN). Colgel has been shown to reduce postoperative bleeding in cardiac surgery [104], while CollaTape and CollaPlug have been used to achieve hemostasis in oral extractions, grafts, and wound sites [178]. These products—except Colgel®—were approved by FDA [107–109]. Microfibrillar collagen has been reported to cause granuloma formation at the site of use [179], and prion disease is always a theoretical concern with the use of bovine products [110].

CoStasis® (Cohesion Technologies, Palo Alto, CA) is another formulation of microfibrillar collagen, composed of bovine thrombin and bovine collagen matrix. Intraoperatively, equal parts of CoStasis and autologous plasma are combined and administered as a sprayable liquid, creating a substrate loaded with thrombin which then cleaves the patient’s fibrinogen, leading to clot formation [111]. It showed improved hemostasis as compared to manual compression in orthopedic, general, hepatic, and cardiac operations [111]. After a clinical study comparing 167 patients treated with CoStasis and 151 controls showed increased efficacy of CoStasis in obtaining hemostasis over standard methods, it was recommended to be used in difficult-to-manage hemorrhage [111].

#### Biological hemostats

Topical thrombin liquid (spray) including bovine origin and human origin have been tested as hemostats biomaterial. Recombinant human thrombin is a promising candidate because it reduced bleeding and thrombotic complications while being less immunogenic than bovine thrombin [121]. Bovine thrombin products such as Thrombin-JMI® (Pfizer) and Thrombogen™ (Johnson & Johnson) are not approved by FDA because of the immunogenic effects in human. Human thrombin products approved by FDA include Evithrom® (OMRIX Biopharmaceuticals, Inc., New York City, NY) and Recothrom® (ZymoGenetics, Inc. Seattle, WA) [123, 124]. The application of thrombin is usually combined with other substrates, such as a damp sponge or gelatin sponge, to apply a thrombin solution to the wound site. It has been shown to be an effective hemostat in comparative trials in liver resection, spinal surgery, peripheral arterial bypass, and dialysis access surgery [121]. However, pooled human thrombin raises infectious disease concerns [29], while purified bovine thrombin activates autoimmune antibodies—typically against factor V—potentially causing profound coagulopathy [122].

More recently (2018 and 2019), it was found that the use of platelet-rich fibrin membranes may represent a feasible alternative hemostatic material for the treatment of oral lesions [126, 180]. Platelets acts as autologous sources of cytokines and growth factors to treat hemorrhage. Platelet concentrate derived from blood can be used to prevent and treat bleeding due to severe thrombocytopenia and oral hemorrhage associated with medullary aplasia, acute leukemia [181]. Because of the role of platelets in coagulation, platelet-rich membranes might have a promising application in post-tonsillectomy hemorrhage.

Chitin is a natural substance found in the exoskeletons of arthropods, the cell walls of some fungi, or as a byproduct of algae fermentation. Chitin and its derivative chitosan have myriad beneficial effects on hemostasis and wound healing. Chitin and chitosan facilitate hemostasis by providing a scaffold for erythrocyte aggregation, increasing platelet and clotting factor delivery to the wound, and by promoting local vasoconstriction [29, 178]. Further, both have been shown to improve the speed of wound healing and reconstruction of connective tissue, in addition to having painkilling and antimicrobial properties [182–186]. Formulations are gels, fibers, films, or beads, with many options for application in the oral cavity [29]. Biodegradation studies have shown persistence of chitosan at wound sites up to 14 days after administration, with minimal local or systemic risks [187]. ChitoFlex® and HemCon® (HemCon Medical Technologies Inc., Portland, OR), Celox™ (SAM Medical Products, Portland, OR) are commercial chitin/chitosan products. Novel chitosan delivery methods, such as polyvinylpyrrolidone-chitosan film and chitosan-alginate hydrogel, are in development to be applicable in the oral cavity [186, 188]. Chitin and chitosan represent one of the most promising materials that has yet to be directly studied in PTH prevention.

Additional biomaterials listed in Table 2 include Hemocoagulase agkistrodon (Konruns Pharmaceutical Co., Ltd., China), zeolite-based QuikClot® (Z-Medica Corp, Wallingford, CT) and alginate dressing Silverlon™ Antimicrobial Calcium Alginate Dressing (Argentum Medical, LLC, Geneva, IL). They have been applied in oral and cardiac surgeries, and proved effective in hemostasis and wound healing. But each has limitations: profound coagulopathy by topical thrombin liquid, handling difficulty of platelet-rich fibrin membranes, and lack of substrate for chitin/chitosan dressing. Addressing these limitations will promote an immediately apparent utility in PTH prevention.

#### Tissue adhesives

Tissue adhesives have been widely used to provide hemostasis for PTH (fibrin-based sealant in Table 1). Other types of tissue adhesives (Table 2) have been applied as hemostats in oral and maxillofacial surgeries [29]. The commercial products of the listed tissue adhesives are 1) Cyanoacrylates: Omnex® Surgical Sealant (Ethicon); 2) polyethylene glycol hydrogel: CoSeal® (Baxter International) and DuraSeal® (Integra lifesciences, Plainsboro, NJ); and 3) Mixture of bovine serum albumin (BSA) and glutaraldehyde: Bioglue® (CryoLife Inc., Atlanta, GA). Methyl cellulose based mucosal tissue dressing [153] and nature polymer based tissue adhesive [154] are patent describing desirable adhesive properties for reducing post-operative pain and bleeding, and promoting wound healing. Information regarding the application, advantages, drawbacks, and FDA approval status are listed in Table 2. Any biomaterial that would be used for PTH prevention would be exposed to consistent shear forces and a complex environment as soon as applied to the wound site in the oropharynx. The adhesive biomaterials provide a mechanical barrier to protect tonsil wound from friction by food in the first few days after surgery, thus enhance healing after tonsillectomy. Thus high adhesive strength is one of the most required properties for PTH that one biomaterial should be manufactured with.

## Conclusion

The lack of widespread clinical adoption of both current and potential biomaterials is due to the following difficulties in PTH prevention: 1) The vascular supply to the tonsil is robust, making tonsillectomy the “ultimate test of hemostasis”; 2) The complex environment is routinely exposed to saliva and bacteria; 3) Shear forces from swallowing (food viscosity) and coughing (high flow rate) make tissue adhesion challenging. These three criteria can be used as an a priori assessment of the potential utility of any biomaterial in PTH prevention.

Any biomaterial that would be used in the prevention of PTH should be biocompatible, easy to apply, cost efficient, reduce complications, remain stable as protective physical barrier, and promote wound healing and remucosalization. Among the reviewed current biomaterials for PTH, the fibrin-based collagen fleece and gelatin-based patch are more effective for PTH than the other biomaterials. In alternative biomaterials, tissue adhesives and chitin/chitosan materials seem promising to be tested for PTH, because their adhesive properties might benefit hemostasis in the tonsil environment. The most common mechanism of action utilized to prevent PTH is formation of the fibrin clot which improves wound healing and hemostasis. For the developing biomaterials to prevent PTH, it is necessary to perform comparative clinical trials to elucidate their capacities to achieve hemostasis, their biocompatibility, and their adhesive properties.

## Data Availability

Not applicable.

## Abbreviations

BSA: Bovine serum albumin
BSG: Bismuth subgallate
FDA: Food and drug administration
GTHM: Gelatin-thrombin hemostatic matrix
MMS: Microporous mucopolysaccharide spheres
MPH: Microporous polysaccharide hemispheres
N2O4: Nitrogen tetroxides
PTH: Post-tonsillectomy hemorrhage
TXA: Tranexamic acid
